# Poly[[diaqua­tris[μ_4_-(*p*-phenyl­enedi­oxy)diacetato]didysprosium(III)] dihydrate]

**DOI:** 10.1107/S1600536808017649

**Published:** 2008-06-19

**Authors:** Ya-Feng Li, Dao-Wu Wang, Yuan-Rui Wang, Long Zhang

**Affiliations:** a School of Chemical Engineering, Changchun University of Technology, Changchun 130012, People’s Republic of China

## Abstract

The title dysprosium coordination polymer, {[Dy_2_(C_10_H_8_O_6_)_3_(H_2_O)_2_]·2H_2_O}_*n*_, was synthesized by reacting dysprosium(III) nitrate and the flexible ligand (*p*-phenyl­enedi­oxy)diacetic acid under hydro­thermal conditions. Each Dy^III^ ion is coordinated by nine O atoms in a tricapped trigonal prismatic geometry. The DyO_9_ polyhedra form layers parallel to the *bc* plane. The rigid benzene rings of the anions link the layers along the *a* axis, forming a three-dimensional framework.

## Related literature

For related literature, see: Eddaoudi *et al.* (2001 ▶); Li & Han (2006 ▶); Michl (1995 ▶); Yaghi *et al.* (1998 ▶). 
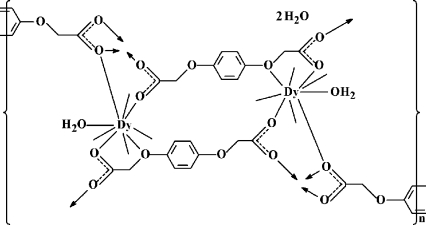


## Experimental

### 

#### Crystal data

[Dy_2_(C_10_H_8_O_6_)_3_(H_2_O)_2_]·2H_2_O
*M* *_r_* = 1069.56
Monoclinic, 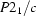

*a* = 12.080 (2) Å
*b* = 16.615 (3) Å
*c* = 8.8802 (18) Å
β = 109.32 (3)°
*V* = 1682.0 (6) Å^3^
*Z* = 2
Mo *K*α radiation
μ = 4.50 mm^−1^
*T* = 293 (2) K
0.19 × 0.16 × 0.13 mm

#### Data collection

Rigaku R-AXIS RAPID diffractometer
Absorption correction: multi-scan (*ABSCOR*; Higashi, 1995 ▶) *T*_min_ = 0.437, *T*_max_ = 0.548
16183 measured reflections
3838 independent reflections
3229 reflections with *I* > 2σ(*I*)
*R*_int_ = 0.043

#### Refinement

*R*[*F* ^2^ > 2σ(*F* ^2^)] = 0.028
*wR*(*F* ^2^) = 0.059
*S* = 1.05
3838 reflections
256 parameters
6 restraints
H atoms treated by a mixture of independent and constrained refinement
Δρ_max_ = 0.83 e Å^−3^
Δρ_min_ = −0.93 e Å^−3^

### 

Data collection: *PROCESS-AUTO* (Rigaku, 1998 ▶); cell refinement: *PROCESS-AUTO*; data reduction: *CrystalStructure* (Rigaku/MSC, 2002 ▶); program(s) used to solve structure: *SHELXS97* (Sheldrick, 2008 ▶); program(s) used to refine structure: *SHELXL97* (Sheldrick, 2008 ▶); molecular graphics: *DIAMOND* (Brandenburg, 2000 ▶); software used to prepare material for publication: *SHELXL97*.

## Supplementary Material

Crystal structure: contains datablocks I, global. DOI: 10.1107/S1600536808017649/ci2601sup1.cif

Structure factors: contains datablocks I. DOI: 10.1107/S1600536808017649/ci2601Isup2.hkl

Additional supplementary materials:  crystallographic information; 3D view; checkCIF report

## Figures and Tables

**Table 1:** Hydrogen-bond geometry (Å, °)

*D*—H⋯*A*	*D*—H	H⋯*A*	*D*⋯*A*	*D*—H⋯*A*
O1*W*—H16*A*⋯O6^i^	0.80 (3)	2.11 (5)	2.706 (4)	131 (6)
O1*W*—H16*B*⋯O2*W*	0.79 (3)	1.98 (4)	2.755 (6)	166 (7)
O2*W*—H17*A*⋯O3	0.82 (3)	2.41 (6)	2.952 (5)	125 (5)

Symmetry code: (i) 
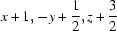
.

**Fig. 1.**
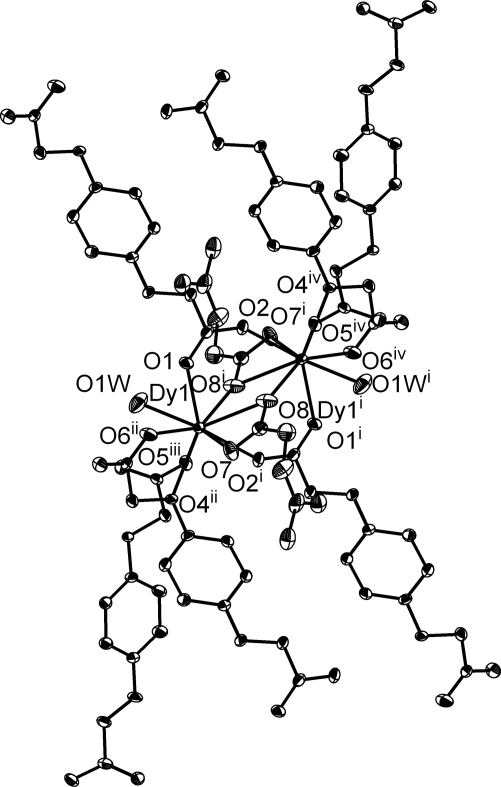
The dinuclear unit of the title polymeric complex, showing the atomic labelling and displacement ellipsoids at the 50% probability level. H atoms have been omitted for clarity [symmetry codes: (i) -x, -y, -z; (ii) 1 + x, y, 1 + z; (iii) 1 + x, 1/2 - y, 3/2 + z; (iv) -1 - x, -y, -1 - z.]

**Fig. 2.**
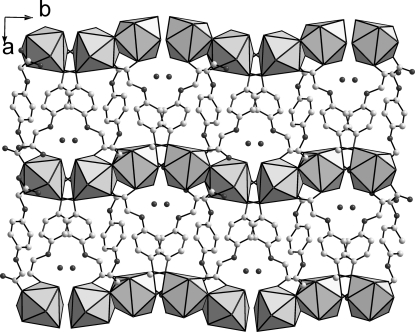
A polyhedral and ball-stick plot of three-dimensional network in the complex, viewed along the c axis. O atoms are shown in dark gray and C atoms in light gray. H atoms have been omitted for clarity.

## supplementary crystallographic information

### Comment

Extensive efforts have been made to construct metal-organic frameworks (MOFs) owing to the benefits they offer such as functional mesoscopic phases, modified surfaces and designed crystals (Michl, 1995). The carboxylate group can offer more solid frameworks by forming M—O—C cluster. The recent works focus on metals and rigid multidentate phenylcarboxylates to form MOFs (Yaghi *et al.*, 1998; Eddaoudi *et al.*, 2001). The chemistry of rare earth compounds is always an attactive research direction due to their complicated geometry and many important applications. We have constructed a MOF by using a rare earth and a flexible multidentate carboxylate ligand with rigid phenyl core, and its crystal structure is reported here.

The asymmetric unit of the title compound contains one Dy atom, one and half (p-phenylenedioxy)diacetate (BDOA) anions and two water molecules. One of the anions is loacted in a general position (say BDOA-N) and the other (say BDOA-C) lies on an inversion center. Each Dy^III^ cation is coordinated by two BDOA-C anions and four BDOA-N anions (Fig.1). The BDOA-C anion provides O atom from the carboxylate group, while the O atoms provided by the BDOA-N anions are from both carboxylate and phenol groups. Each Dy^III^ ion is coordinated by nine O atoms; seven out of the nine O atoms are from carboxylate groups of six BDOA anions, one from the phenolic O atom of one BDOA anion and one from a coordinated water molecule. The coordination environment around the Dy^III^ ion may be described as tricapped trigonal-prismatic, with Dy—O distances in the range 2.333 (10) Å-2.829 (4) Å and O—Dy—O angles in the range 47.86 (10)°-148.5 (1)°. A pair of Dy^III^ atoms are bridged by the two ligands in different coordination modes (Fig. 1). The Dy···Dy distance is 4.1404 (9) Å.

The adjacent DyO_9_ polyhedra form edge-shared dimer, with four BDOA anions. The edge-shared DyO_9_ polyhedra are connected to form layers parallel to the *bc* plane. The rigid phenyl cores of BDOA anions link the layers along the *a* axis to form a three-dimensional framework (Fig.2). The uncoordinated water molecules are trapped inside the channel formed by the DyO_9_ polyhedra and phenyl core of the BDOA anions.

O—H···O hydrogen bonds are observed in the crystal structure (Table 1). The crystal structure of the title compound is similar to that of [La_2_(1,4-BDOA)_3_(H_2_O)]_2_]^.^2H_2_O (Li *et al.*, 2006).

### Experimental

1,4-BDOA (0.30 g, 1.33 mmol) and Dy(NO_3_)_3_^.^6H_2_O (0.30 g, 0.67 mmol) were successively dissolved in aquous NaOH (0.13 g, 3.3 mmol NaOH/7.5 ml distilled water). The molar ratio of Dy(NO_3_)_3_^.^6H_2_O: 1,4-BDOA: NaOH: H_2_O was 1:2:5:630. The solution was continuously stirred for 1 h at room temperature. Finally, the solution was sealed into 23 ml autoclave and was heated at 438 K for 6 d and then naturally cooled to room temperature, to obtain colourless crystals of the title compound.

### Refinement

Water H atoms were located in a difference Fourier map and were refined with O-H = 0.84 (1) Å, H···H = 1.37 (2) Å and *U*_iso_(H) = 1.5*U*_eq_(O). The remaining H-atoms were placed in calculated positions (C-H = 0.93-0.97 Å) and were included in the refinement in the riding-model approximation, with *U*(H) = 1.2*U*_eq_(C).

### Crystal data

**Table:**

[Dy_2_(C_10_H_8_O_6_)_3_(H_2_O)_2_]·2H_2_O	*F*_000_ = 1040
*M**_r_* = 1069.56	*D*_x_ = 2.112 Mg m^−^^3^
Monoclinic, *P*2_1_/*c*	Mo *K*α radiation λ = 0.71073 Å
Hall symbol: -P 2ybc	Cell parameters from 2000 reflections
*a* = 12.080 (2) Å	θ = 3.0–27.5º
*b* = 16.615 (3) Å	µ = 4.50 mm^−^^1^
*c* = 8.8802 (18) Å	*T* = 293 (2) K
β = 109.32 (3)º	Block, grey yellow
*V* = 1682.0 (6) Å^3^	0.19 × 0.16 × 0.13 mm
*Z* = 2	

### Data collection

**Table:**

Rigaku R-AXIS RAPID diffractometer	3838 independent reflections
Radiation source: fine-focus sealed tube	3229 reflections with *I* > 2σ(*I*)
Monochromator: graphite	*R*_int_ = 0.043
Detector resolution: 10.00 pixels mm^-1^	θ_max_ = 27.5º
*T* = 293(2) K	θ_min_ = 3.0º
ω scans	*h* = −15→15
Absorption correction: multi-scan(ABSCOR; Higashi, 1995)	*k* = −21→21
*T*_min_ = 0.437, *T*_max_ = 0.548	*l* = −11→11
16183 measured reflections	

### Refinement

**Table:**

Refinement on *F*^2^	Secondary atom site location: difference Fourier map
Least-squares matrix: full	Hydrogen site location: inferred from neighbouring sites
*R*[*F*^2^ > 2σ(*F*^2^)] = 0.028	H atoms treated by a mixture of independent and constrained refinement
*wR*(*F*^2^) = 0.059	*w* = 1/[σ^2^(*F*_o_^2^) + (0.0147*P*)^2^ + 5.1969*P*] where *P* = (*F*_o_^2^ + 2*F*_c_^2^)/3
*S* = 1.06	(Δ/σ)_max_ = 0.009
3838 reflections	Δρ_max_ = 0.83 e Å^−^^3^
256 parameters	Δρ_min_ = −0.93 e Å^−^^3^
6 restraints	Extinction correction: none
Primary atom site location: structure-invariant direct methods	

### Special details

**Table:**

Geometry. All e.s.d.'s (except the e.s.d. in the dihedral angle between two l.s. planes) are estimated using the full covariance matrix. The cell e.s.d.'s are taken into account individually in the estimation of e.s.d.'s in distances, angles and torsion angles; correlations between e.s.d.'s in cell parameters are only used when they are defined by crystal symmetry. An approximate (isotropic) treatment of cell e.s.d.'s is used for estimating e.s.d.'s involving l.s. planes.
Refinement. Refinement of *F*^2^ against ALL reflections. The weighted *R*-factor *wR* and goodness of fit *S* are based on *F*^2^, conventional *R*-factors *R* are based on *F*, with *F* set to zero for negative *F*^2^. The threshold expression of *F*^2^ > σ(*F*^2^) is used only for calculating *R*-factors(gt) *etc*. and is not relevant to the choice of reflections for refinement. *R*-factors based on *F*^2^ are statistically about twice as large as those based on *F*, and *R*- factors based on ALL data will be even larger.

### Fractional atomic coordinates and isotropic or equivalent isotropic displacement parameters (Å^2^)

**Table:**

	*x*	*y*	*z*	*U*_iso_*/*U*_eq_	
Dy1	0.047406 (15)	0.117791 (10)	0.06685 (2)	0.01642 (6)	
O1	−0.1292 (2)	0.11347 (17)	−0.1593 (3)	0.0272 (7)	
O1W	−0.0933 (3)	0.2099 (2)	0.1170 (4)	0.0492 (11)	
H16A	−0.051 (5)	0.246 (3)	0.161 (7)	0.074*	
H16B	−0.145 (4)	0.228 (3)	0.046 (6)	0.074*	
O2	−0.1802 (2)	−0.01721 (17)	−0.1976 (3)	0.0255 (6)	
O3	−0.3483 (2)	0.15102 (17)	−0.3735 (3)	0.0252 (6)	
O4	−0.7742 (2)	0.21741 (16)	−0.8419 (3)	0.0214 (6)	
O5	−0.8998 (3)	0.35594 (17)	−1.1500 (3)	0.0244 (6)	
O6	−0.9551 (3)	0.23500 (18)	−1.0955 (4)	0.0335 (8)	
O7	0.1721 (3)	0.09109 (18)	−0.0991 (4)	0.0312 (7)	
O8	0.0580 (3)	−0.0133 (2)	−0.1313 (4)	0.0362 (8)	
O9	0.3019 (3)	0.0217 (2)	−0.2669 (4)	0.0417 (9)	
C1	−0.1940 (3)	0.0565 (2)	−0.2298 (5)	0.0199 (8)	
C2	−0.3012 (3)	0.0727 (2)	−0.3743 (5)	0.0245 (9)	
H2A	−0.2803	0.0664	−0.4701	0.029*	
H2B	−0.3611	0.0331	−0.3778	0.029*	
C3	−0.4522 (3)	0.1666 (2)	−0.4950 (5)	0.0193 (8)	
C4	−0.5023 (3)	0.1140 (2)	−0.6214 (4)	0.0211 (8)	
H4	−0.4650	0.0660	−0.6286	0.025*	
C5	−0.5082 (4)	0.2383 (3)	−0.4866 (5)	0.0254 (9)	
H5	−0.4742	0.2739	−0.4033	0.030*	
C6	−0.6087 (3)	0.1338 (2)	−0.7369 (5)	0.0216 (8)	
H6	−0.6418	0.0991	−0.8222	0.026*	
C7	−0.6154 (4)	0.2576 (3)	−0.6023 (5)	0.0242 (9)	
H7	−0.6530	0.3058	−0.5967	0.029*	
C8	−0.6648 (3)	0.2037 (2)	−0.7256 (4)	0.0176 (8)	
C9	−0.7965 (3)	0.2992 (2)	−0.9002 (5)	0.0210 (8)	
H9A	−0.7255	0.3229	−0.9092	0.025*	
H9B	−0.8222	0.3317	−0.8272	0.025*	
C10	−0.8909 (3)	0.2962 (2)	−1.0620 (4)	0.0179 (8)	
C11	0.1401 (3)	0.0239 (3)	−0.1581 (5)	0.0242 (9)	
C12	0.1937 (4)	−0.0131 (3)	−0.2737 (6)	0.0397 (12)	
H12A	0.2054	−0.0702	−0.2510	0.048*	
H12B	0.1389	−0.0075	−0.3812	0.048*	
C13	0.3980 (4)	0.0092 (3)	−0.1300 (6)	0.0340 (11)	
C14	0.4975 (4)	0.0529 (3)	−0.1204 (6)	0.0361 (11)	
H14	0.4960	0.0887	−0.2014	0.043*	
C15	0.4008 (4)	−0.0437 (3)	−0.0085 (6)	0.0357 (11)	
H15	0.3342	−0.0731	−0.0135	0.043*	
O2W	−0.2446 (4)	0.2835 (3)	−0.1500 (5)	0.0604 (12)	
H17A	−0.229 (6)	0.258 (4)	−0.219 (7)	0.091*	
H17B	−0.303 (5)	0.260 (4)	−0.133 (8)	0.091*	

### Atomic displacement parameters (Å^2^)

**Table:**

	*U*^11^	*U*^22^	*U*^33^	*U*^12^	*U*^13^	*U*^23^
Dy1	0.01428 (9)	0.01573 (9)	0.01598 (9)	0.00025 (7)	0.00061 (6)	0.00046 (7)
O1	0.0256 (15)	0.0200 (14)	0.0229 (14)	−0.0024 (12)	−0.0097 (12)	−0.0005 (12)
O1W	0.044 (2)	0.060 (3)	0.0311 (19)	0.0266 (19)	−0.0044 (16)	−0.0118 (18)
O2	0.0257 (15)	0.0170 (14)	0.0278 (15)	0.0069 (11)	0.0010 (13)	0.0013 (12)
O3	0.0199 (14)	0.0197 (14)	0.0236 (14)	0.0063 (11)	−0.0093 (12)	−0.0046 (12)
O4	0.0150 (13)	0.0162 (14)	0.0233 (14)	−0.0012 (10)	−0.0068 (11)	0.0050 (11)
O5	0.0303 (15)	0.0203 (14)	0.0180 (13)	−0.0027 (12)	0.0018 (12)	0.0078 (12)
O6	0.0304 (17)	0.0256 (17)	0.0284 (16)	−0.0090 (13)	−0.0118 (13)	0.0125 (13)
O7	0.0436 (19)	0.0238 (16)	0.0336 (17)	−0.0028 (14)	0.0226 (15)	−0.0064 (13)
O8	0.0270 (16)	0.046 (2)	0.0300 (16)	−0.0127 (15)	0.0015 (14)	0.0092 (15)
O9	0.0338 (18)	0.063 (2)	0.0360 (18)	0.0116 (17)	0.0216 (16)	0.0022 (17)
C1	0.0173 (18)	0.022 (2)	0.0179 (18)	0.0061 (15)	0.0022 (15)	−0.0016 (16)
C2	0.021 (2)	0.018 (2)	0.026 (2)	0.0061 (16)	−0.0042 (17)	−0.0026 (17)
C3	0.0142 (17)	0.019 (2)	0.0204 (18)	0.0005 (14)	0.0000 (15)	0.0004 (15)
C4	0.0196 (18)	0.0181 (19)	0.0205 (18)	0.0024 (16)	−0.0003 (15)	−0.0044 (16)
C5	0.023 (2)	0.023 (2)	0.021 (2)	0.0029 (17)	−0.0057 (17)	−0.0077 (17)
C6	0.0202 (18)	0.017 (2)	0.0211 (19)	−0.0009 (15)	−0.0024 (16)	−0.0027 (15)
C7	0.021 (2)	0.020 (2)	0.026 (2)	0.0051 (16)	0.0002 (17)	−0.0025 (17)
C8	0.0128 (17)	0.0201 (19)	0.0166 (18)	0.0003 (14)	0.0003 (15)	0.0046 (15)
C9	0.024 (2)	0.0146 (19)	0.0195 (19)	0.0001 (15)	0.0008 (16)	0.0022 (15)
C10	0.0168 (18)	0.0158 (19)	0.0178 (18)	0.0027 (14)	0.0013 (15)	0.0026 (15)
C11	0.020 (2)	0.026 (2)	0.027 (2)	−0.0014 (16)	0.0099 (17)	0.0060 (18)
C12	0.036 (3)	0.043 (3)	0.043 (3)	0.001 (2)	0.017 (2)	−0.014 (2)
C13	0.032 (2)	0.036 (3)	0.044 (3)	0.011 (2)	0.025 (2)	−0.001 (2)
C14	0.043 (3)	0.032 (3)	0.047 (3)	0.009 (2)	0.032 (2)	0.009 (2)
C15	0.033 (2)	0.033 (3)	0.050 (3)	0.003 (2)	0.026 (2)	0.001 (2)
O2W	0.055 (3)	0.060 (3)	0.051 (3)	0.016 (2)	−0.003 (2)	−0.013 (2)

### Geometric parameters (Å, °)

**Table:**

Dy1—O8^i^	2.333 (3)	C3—O3	1.382 (4)
Dy1—O2^i^	2.341 (3)	C3—C5	1.385 (5)
Dy1—O1	2.399 (3)	C3—C4	1.392 (5)
Dy1—O6^ii^	2.417 (3)	C4—C6	1.392 (5)
Dy1—O5^iii^	2.421 (3)	C4—H4	0.93
Dy1—O1W	2.435 (4)	C5—C7	1.399 (5)
Dy1—O7	2.471 (3)	C5—H5	0.93
Dy1—O4^ii^	2.624 (3)	C6—C8	1.366 (5)
Dy1—O8	2.830 (4)	C6—H6	0.93
O1—C1	1.255 (5)	C7—C8	1.387 (5)
O1W—H16A	0.80 (3)	C7—H7	0.93
O1W—H16B	0.79 (3)	C9—C10	1.512 (5)
O2—C1	1.256 (5)	C9—H9A	0.97
O3—C3	1.382 (4)	C9—H9B	0.97
O3—C2	1.421 (5)	C11—C12	1.514 (6)
O4—C8	1.400 (4)	C12—H12A	0.97
O4—C9	1.448 (4)	C12—H12B	0.97
O5—C10	1.246 (5)	C13—C14	1.382 (7)
O6—C10	1.253 (5)	C13—C15	1.384 (7)
O7—C11	1.240 (5)	C14—C15^iv^	1.383 (7)
O8—C11	1.257 (5)	C14—H14	0.93
O9—C13	1.391 (6)	C15—C14^iv^	1.383 (7)
O9—C12	1.413 (6)	C15—H15	0.93
C1—C2	1.516 (5)	O2W—H17A	0.82 (3)
C2—H2A	0.97	O2W—H17B	0.86 (3)
C2—H2B	0.97		
			
O8^i^—Dy1—O2^i^	71.77 (11)	O3—C2—C1	113.0 (3)
O8^i^—Dy1—O1	77.10 (10)	O3—C2—H2A	109.0
O2^i^—Dy1—O1	131.87 (10)	C1—C2—H2A	109.0
O8^i^—Dy1—O6^ii^	147.73 (10)	O3—C2—H2B	109.0
O2^i^—Dy1—O6^ii^	137.92 (11)	C1—C2—H2B	109.0
O1—Dy1—O6^ii^	72.10 (10)	H2A—C2—H2B	107.8
O8^i^—Dy1—O5^iii^	81.77 (11)	O3—C3—C5	116.9 (3)
O2^i^—Dy1—O5^iii^	73.24 (10)	O3—C3—C5	116.9 (3)
O1—Dy1—O5^iii^	136.95 (11)	O3—C3—C4	123.6 (3)
O6^ii^—Dy1—O5^iii^	114.92 (10)	O3—C3—C4	123.6 (3)
O8^i^—Dy1—O1W	87.26 (14)	C5—C3—C4	119.5 (3)
O2^i^—Dy1—O1W	139.73 (12)	C3—C4—C6	119.7 (4)
O1—Dy1—O1W	71.98 (11)	C3—C4—H4	120.1
O6^ii^—Dy1—O1W	74.39 (14)	C6—C4—H4	120.1
O5^iii^—Dy1—O1W	69.93 (11)	C3—C5—C7	120.5 (4)
O8^i^—Dy1—O7	120.17 (11)	C3—C5—H5	119.7
O2^i^—Dy1—O7	73.43 (11)	C7—C5—H5	119.7
O1—Dy1—O7	92.54 (11)	C8—C6—C4	120.4 (4)
O6^ii^—Dy1—O7	71.26 (11)	C8—C6—H6	119.8
O5^iii^—Dy1—O7	130.44 (10)	C4—C6—H6	119.8
O1W—Dy1—O7	145.27 (13)	C8—C7—C5	119.0 (4)
O8^i^—Dy1—O4^ii^	148.49 (9)	C8—C7—H7	120.5
O2^i^—Dy1—O4^ii^	86.51 (9)	C5—C7—H7	120.5
O1—Dy1—O4^ii^	133.60 (9)	C6—C8—C7	120.8 (3)
O6^ii^—Dy1—O4^ii^	61.51 (9)	C6—C8—O4	117.0 (3)
O5^iii^—Dy1—O4^ii^	70.01 (10)	C7—C8—O4	122.2 (3)
O1W—Dy1—O4^ii^	95.66 (12)	O4—C9—C10	107.5 (3)
O7—Dy1—O4^ii^	72.37 (10)	O4—C9—H9A	110.2
O8^i^—Dy1—O8	73.76 (13)	C10—C9—H9A	110.2
O2^i^—Dy1—O8	66.07 (10)	O4—C9—H9B	110.2
O1—Dy1—O8	70.55 (10)	C10—C9—H9B	110.2
O6^ii^—Dy1—O8	104.10 (10)	H9A—C9—H9B	108.5
O5^iii^—Dy1—O8	137.24 (9)	O5—C10—O6	125.5 (3)
O1W—Dy1—O8	140.83 (11)	O5—C10—C9	116.8 (3)
O7—Dy1—O8	47.87 (9)	O6—C10—C9	117.8 (3)
O4^ii^—Dy1—O8	118.45 (9)	O7—C11—O8	121.3 (4)
C1—O1—Dy1	132.4 (2)	O7—C11—C12	120.6 (4)
Dy1—O1W—H16A	101 (5)	O8—C11—C12	118.0 (4)
Dy1—O1W—H16B	121 (5)	O9—C12—C11	113.7 (4)
H16A—O1W—H16B	108 (4)	O9—C12—H12A	108.8
C1—O2—Dy1^i^	145.7 (3)	C11—C12—H12A	108.8
C3—O3—C2	115.4 (3)	O9—C12—H12B	108.8
C8—O4—C9	115.7 (3)	C11—C12—H12B	108.8
C8—O4—Dy1^v^	127.1 (2)	H12A—C12—H12B	107.7
C9—O4—Dy1^v^	116.5 (2)	C14—C13—C15	119.3 (5)
C10—O5—Dy1^vi^	137.2 (3)	C14—C13—O9	115.6 (4)
C10—O6—Dy1^v^	128.9 (2)	C15—C13—O9	125.0 (5)
C11—O7—Dy1	104.3 (3)	C13—C14—C15^iv^	120.6 (4)
C11—O8—Dy1^i^	160.3 (3)	C13—C14—H14	119.7
C11—O8—Dy1	86.5 (3)	C15^iv^—C14—H14	119.7
Dy1^i^—O8—Dy1	106.24 (12)	C14^iv^—C15—C13	120.0 (5)
C13—O9—C12	118.0 (4)	C14^iv^—C15—H15	120.0
O1—C1—O2	127.4 (3)	C13—C15—H15	120.0
O1—C1—C2	120.3 (3)	H17A—O2W—H17B	108 (4)
O2—C1—C2	112.2 (3)		
			
O8^i^—Dy1—O1—C1	−35.1 (4)	C2—O3—C3—C5	172.1 (4)
O2^i^—Dy1—O1—C1	15.4 (4)	O3—O3—C3—C4	0.0 (3)
O6^ii^—Dy1—O1—C1	154.7 (4)	C2—O3—C3—C4	−7.1 (6)
O5^iii^—Dy1—O1—C1	−97.7 (4)	O3—C3—C4—C6	178.5 (4)
O1W—Dy1—O1—C1	−126.4 (4)	O3—C3—C4—C6	178.5 (4)
O7—Dy1—O1—C1	85.3 (4)	C5—C3—C4—C6	−0.6 (6)
O4^ii^—Dy1—O1—C1	153.2 (3)	O3—C3—C5—C7	−178.1 (4)
O8—Dy1—O1—C1	42.0 (4)	O3—C3—C5—C7	−178.1 (4)
O8^i^—Dy1—O7—C11	14.8 (3)	C4—C3—C5—C7	1.1 (7)
O2^i^—Dy1—O7—C11	71.4 (3)	C3—C4—C6—C8	−0.9 (6)
O1—Dy1—O7—C11	−61.7 (3)	C3—C5—C7—C8	0.1 (7)
O6^ii^—Dy1—O7—C11	−131.9 (3)	C4—C6—C8—C7	2.1 (6)
O5^iii^—Dy1—O7—C11	121.0 (3)	C4—C6—C8—O4	−176.8 (4)
O1W—Dy1—O7—C11	−123.1 (3)	C5—C7—C8—C6	−1.6 (6)
O4^ii^—Dy1—O7—C11	163.0 (3)	C5—C7—C8—O4	177.2 (4)
O8—Dy1—O7—C11	−1.0 (2)	C9—O4—C8—C6	−139.9 (4)
O8^i^—Dy1—O8—C11	−164.8 (3)	Dy1^v^—O4—C8—C6	49.9 (5)
O2^i^—Dy1—O8—C11	−87.9 (2)	C9—O4—C8—C7	41.2 (5)
O1—Dy1—O8—C11	113.5 (3)	Dy1^v^—O4—C8—C7	−129.0 (3)
O6^ii^—Dy1—O8—C11	48.6 (2)	C8—O4—C9—C10	158.0 (3)
O5^iii^—Dy1—O8—C11	−107.1 (3)	Dy1^v^—O4—C9—C10	−30.8 (4)
O1W—Dy1—O8—C11	131.1 (3)	Dy1^vi^—O5—C10—O6	−34.7 (7)
O7—Dy1—O8—C11	1.0 (2)	Dy1^vi^—O5—C10—C9	146.8 (3)
O4^ii^—Dy1—O8—C11	−16.3 (3)	Dy1^v^—O6—C10—O5	−177.4 (3)
O8^i^—Dy1—O8—Dy1^i^	0.0	Dy1^v^—O6—C10—C9	1.1 (6)
O2^i^—Dy1—O8—Dy1^i^	76.87 (12)	O4—C9—C10—O5	−160.8 (3)
O1—Dy1—O8—Dy1^i^	−81.74 (12)	O4—C9—C10—O6	20.5 (5)
O6^ii^—Dy1—O8—Dy1^i^	−146.60 (11)	Dy1—O7—C11—O8	2.0 (5)
O5^iii^—Dy1—O8—Dy1^i^	57.68 (18)	Dy1—O7—C11—C12	177.9 (3)
O1W—Dy1—O8—Dy1^i^	−64.1 (2)	Dy1^i^—O8—C11—O7	−133.2 (8)
O7—Dy1—O8—Dy1^i^	165.76 (18)	Dy1—O8—C11—O7	−1.7 (4)
O4^ii^—Dy1—O8—Dy1^i^	148.44 (9)	Dy1^i^—O8—C11—C12	50.8 (10)
Dy1—O1—C1—O2	−0.9 (7)	Dy1—O8—C11—C12	−177.7 (4)
Dy1—O1—C1—C2	−179.0 (3)	C13—O9—C12—C11	68.3 (6)
Dy1^i^—O2—C1—O1	−18.9 (8)	O7—C11—C12—O9	18.6 (6)
Dy1^i^—O2—C1—C2	159.4 (4)	O8—C11—C12—O9	−165.4 (4)
O3—O3—C2—C1	0.00 (8)	C12—O9—C13—C14	−172.0 (4)
C3—O3—C2—C1	−174.5 (4)	C12—O9—C13—C15	9.4 (7)
O1—C1—C2—O3	−27.4 (6)	C15—C13—C14—C15^iv^	0.4 (8)
O2—C1—C2—O3	154.2 (4)	O9—C13—C14—C15^iv^	−178.4 (4)
C2—O3—C3—O3	0(43)	C14—C13—C15—C14^iv^	−0.4 (8)
O3—O3—C3—C5	0.00 (12)	O9—C13—C15—C14^iv^	178.3 (4)

Symmetry codes: (i) −*x*, −*y*, −*z*; (ii) *x*+1, *y*, *z*+1; (iii) *x*+1, −*y*+1/2, *z*+3/2;  (iv) −*x*+1, −*y*, −*z*; (v) *x*−1, *y*, *z*−1; (vi) *x*−1, −*y*+1/2, *z*−3/2.

### Hydrogen-bond geometry (Å, °)

**Table:**

*D*—H···*A*	*D*—H	H···*A*	*D*···*A*	*D*—H···*A*
O1W—H16A···O6^iii^	0.80 (3)	2.11 (5)	2.706 (4)	131 (6)
O1W—H16B···O2W	0.79 (3)	1.98 (4)	2.755 (6)	166 (7)
O2W—H17A···O3	0.82 (3)	2.41 (6)	2.952 (5)	125 (5)

Symmetry codes: (iii) *x*+1, −*y*+1/2, *z*+3/2.
